# Supplementary material for: Intermethod Characterization of Commercially Available Extracellular Vesicles as Reference Materials
Source: Biomolecules. 2025 Dec 31;16(1):66. doi: 10.3390/biom16010066 (PMC12838729; doi:10.3390/biom16010066)
Supplement: Supplementary file 1 [file biomolecules-16-00066-s001.zip › biomolecules-3891676-supplementary.pdf]

Supplementary Information

Supplemental Table S1. Summary statistics of PSD and PNC per replicate for GFP VLPs. Instrument or manual size cut-off; the total number of particles counted to calculate uncertainty values.

GFP VLPs		MRPS	AF^4	PTA	Cryo-EM
**Measurement cutoff (nm)**		**65**	**30**	**25**[(a)]	
**Diameter range (nm)**	Replicate 1	65 to 335	110 to 228	27 to 380	
	Replicate 2	65 to 355	123 to 217	24 to 322	
	Replicate 3	65 to 355	90 to 205	39 to 389	
**Mean Diameter (nm)**	Replicate 1	165	203	116	
	Replicate 2	171	194	118	
	Replicate 3	171	181	118	
**Standard Deviation (nm)**	Replicate 1	77	12	35	
	Replicate 2	83	10	36	
	Replicate 3	83	12	35	
**Concentration (1/mL)**	Replicate 1	$3.64\text{x}10^{09}$	$4.0\text{ x}10^{10}$	$1.0\text{ x}10^{10}$	
	Replicate 2	$7.92\text{ x}10^{09}$	$5.0\text{ x}10^{10}$	$9.2\text{ x}10^{09}$	
	Replicate 3	$1.31\text{ x}10^{10}$	$6.5\text{ x}10^{10}$	$8.6\text{ x}10^{09}$	
**Standard Deviation (1/mL)**	Replicate 1	$7.28\text{ x}10^{07}$	$3.5\text{ x}10^{08}$	$7.8\text{ x}10^{08}$	
	Replicate 2	$1.11\text{ x}10^{08}$	$4.6\text{ x}10^{08}$	$1.3\text{ x}10^{09}$	
	Replicate 3	$1.97\text{ x}10^{08}$	$8.0\text{ x}10^{08}$	$9.9\text{ x}10^{08}$	
**Particles Analyzed**	Replicate 1	3380	$2.02\text{ x}10^{09}$	1744	
	Replicate 2	6910	$2.47\text{ x}10^{09}$	1635	
	Replicate 3	5765	$3.26\text{ x}10^{09}$	1619	

(a) Measurement cutoff for PTA is dependent upon sample and instrument settings. Please refer to the Methods and Materials section for PTA setup.

Supplemental Table S2. Summary statistics of PSD and PNC per replicate for LNCaP-derived EVs. Instrument or manual size cut-off; the total number of particles counted to calculate uncertainty values.

LNCaP EVs		MRPS	AF^4	PTA	Cryo-EM
**Measurement Cutoff (nm)**		**65**	**30**	**62**[(a)]	30
**Diameter range (nm)**	Replicate 1	65 to 274	144 to 194	76 to 217	31 to 152
	Replicate 2	65 to 309	148 to 195	62 to 274	
	Replicate 3	65 to 291	156 to 198	74 to 214	
**Mean Diameter (nm)**	Replicate 1	86	177	133	54
	Replicate 2	86	183	127	
	Replicate 3	86	185	129	
**Standard Deviation (nm)**	Replicate 1	23	13	29	17
	Replicate 2	22	11	37	
	Replicate 3	22	9	30	
**Concentration (1/mL)**	Replicate 1	$8.6\text{ x}10^{11}$	$4.6\text{ x}10^{10}$	$3.8\text{ x}10^{12}$	
	Replicate 2	$6.3\text{ x}10^{11}$	$5.0\text{ x}10^{10}$	$3.6\text{ x}10^{12}$	
	Replicate 3	$6.3\text{ x}10^{11}$	$5.1\text{ x}10^{10}$	$2.8\text{ x}10^{12}$	
**Standard Deviation (1/mL)**	Replicate 1	$1.3\text{ x}10^{10}$	$1.3\text{ x}10^{08}$	$4.2\text{ x}10^{11}$	
	Replicate 2	$1.1\text{ x}10^{10}$	$1.5\text{ x}10^{08}$	$5.6\text{ x}10^{11}$	
	Replicate 3	$9.4\text{ x}10^{09}$	$2.8\text{ x}10^{08}$	$8.5\text{ x}10^{11}$	
**Particles Analyzed**	Replicate 1	5990	$2.3\text{ x}10^{09}$	165	511
	Replicate 2	4618	$2.5\text{ x}10^{09}$	117	
	Replicate 3	5846	$2.6\text{ x}10^{09}$	111	

(a) Measurement cutoff for PTA is dependent upon sample and instrument settings. Please refer to the Methods and Materials section for PTA setup.

Supplemental Table S3. Summary statistics of PSD and PNC per replicate for MSC-derived EVs. Instrument or manual size cut-off; the total number of particles counted to calculate uncertainty values.

MSC EVs		MRPS	AF⁴	PTA	Cryo-EM
**Measurement Cut-off (nm)**		**65**	**30**	**42** [(a)]	30
**Diameter range (nm)**	Replicate 1	65 to 321	123 to 169	55 to 300	36 to 182
	Replicate 2	65 to 341	158 to 199	42 to 312	
	Replicate 3	65 to 279	129 to 186	46 to 267	
**Mean Diameter (nm)**	Replicate 1	83	144	118	61
	Replicate 2	74	184	118	
	Replicate 3	80	162	110	
**Standard Deviation (nm)**	Replicate 1	27	7	34	19
	Replicate 2	18	5	38	
	Replicate 3	21	10	35	
**Concentration (1/mL)**	Replicate 1	9.3 $\times10^{11}$	8.6 $\times10^{10}$	2.3 $\times10^{13}$	
	Replicate 2	1.58 $\times10^{12}$	2.8 $\times10^{11}$	1.8 $\times10^{13}$	
	Replicate 3	7.3 $\times10^{11}$	4.6 $\times10^{11}$	1.9 $\times10^{13}$	
**Standard Deviation (1/mL)**	Replicate 1	0.1 $\times10^{11}$	5.4 $\times10^{08}$	9.7 $\times10^{12}$	
	Replicate 2	0.024 $\times10^{12}$	4.1 $\times10^{09}$	4.4 $\times10^{12}$	
	Replicate 3	0.1 $\times10^{11}$	1.0 $\times10^{10}$	2.5 $\times10^{12}$	
**Particles Analyzed**	Replicate 1	3160	5.2 $\times10^{09}$	375	561
	Replicate 2	3291	1.7 $\times10^{10}$	449	
	Replicate 3	2085	2.8 $\times10^{10}$	400	

(a) Measurement cutoff for PTA is dependent upon sample and instrument settings. Please refer to the Methods and Materials section for PTA setup.

Supplemental Table S4. Analysis of small RNA extractions from LNCaP EVs by nanodrop

Small RNA	Conc (ng/uL)	A260/A280	A260/A230
Vial 1	4.3 +/- 0.3	2.4 +/- 0.2	0.033+/- 0.008
Vial 2	9.8 +/- 2	1.7 +/- 0.05	0.74 +/- 0.04

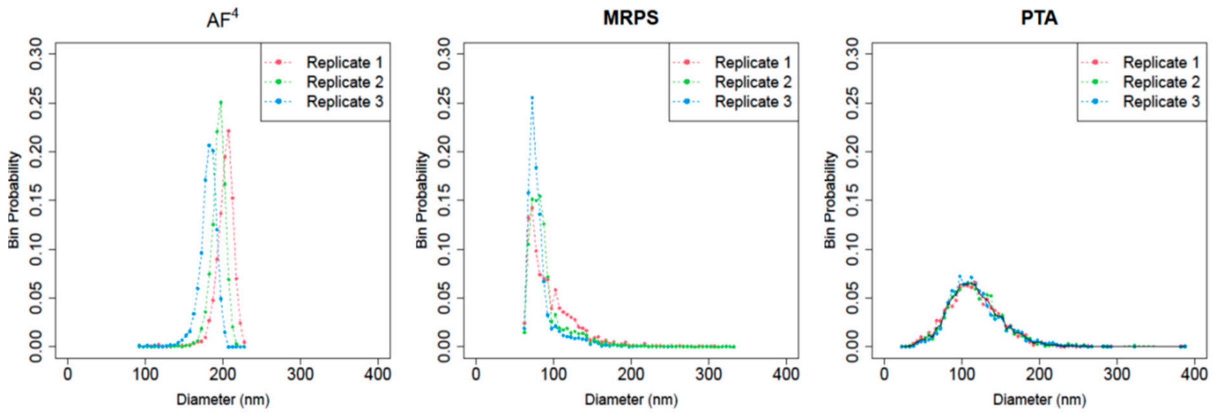

Supplemental Figure S1. PSD for GFP VLPs was measured in triplicate for each method AF[4] (left), MRPS (center), and PTA (right).

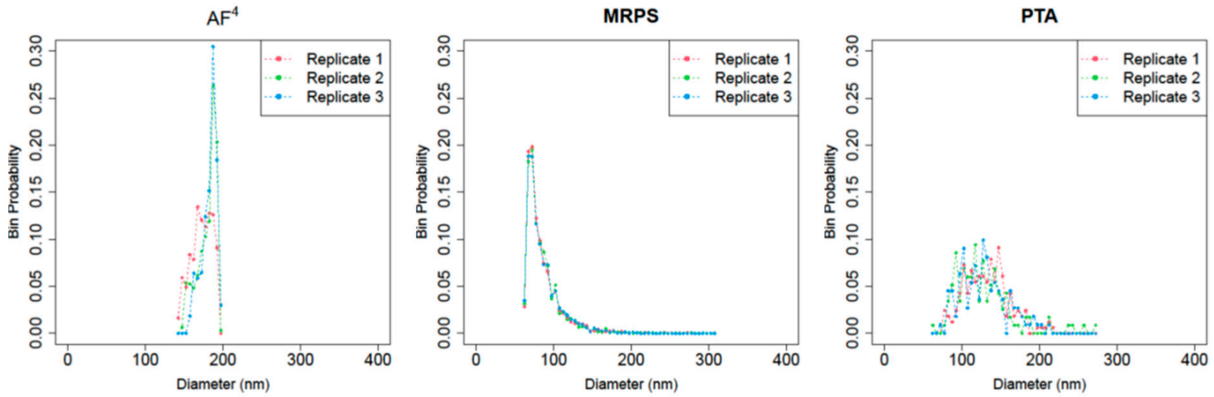

Supplemental Figure S2. PSD for LNCaP EV was measured in triplicate for each method: AF[4] (left), MRPS (center), and PTA (right).

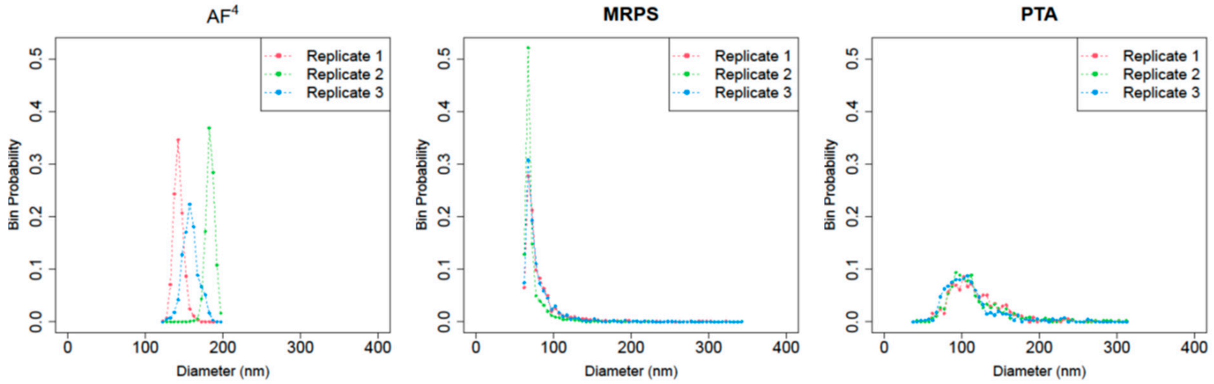

Supplemental Figure S3. PSD for MSC EV was measured in triplicate for each method: AF[4] (left), MRPS (center), and PTA (right).

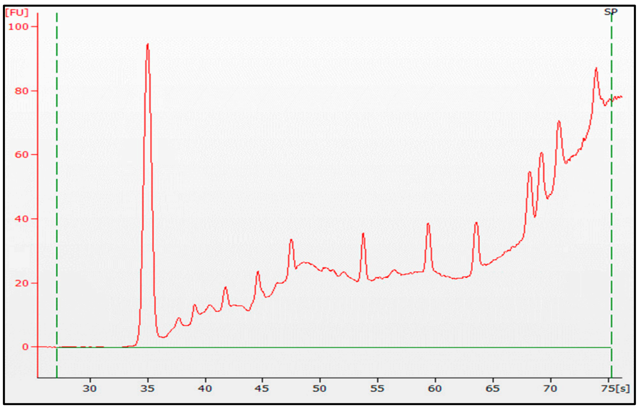

Supplemental Figure S4. Bioanalysis of small RNA extracted from LNCaP EVs; multiple RNA species/peaks were observed during the analysis of small RNA population by Agilent 2100 bioanalyzer, molecular ladders sized 6 nts (first peak around 35s) and 150 nts (last peak around 75s) were used for RNA size estimation. A representative bioanalyzer read from four independent runs is shown.

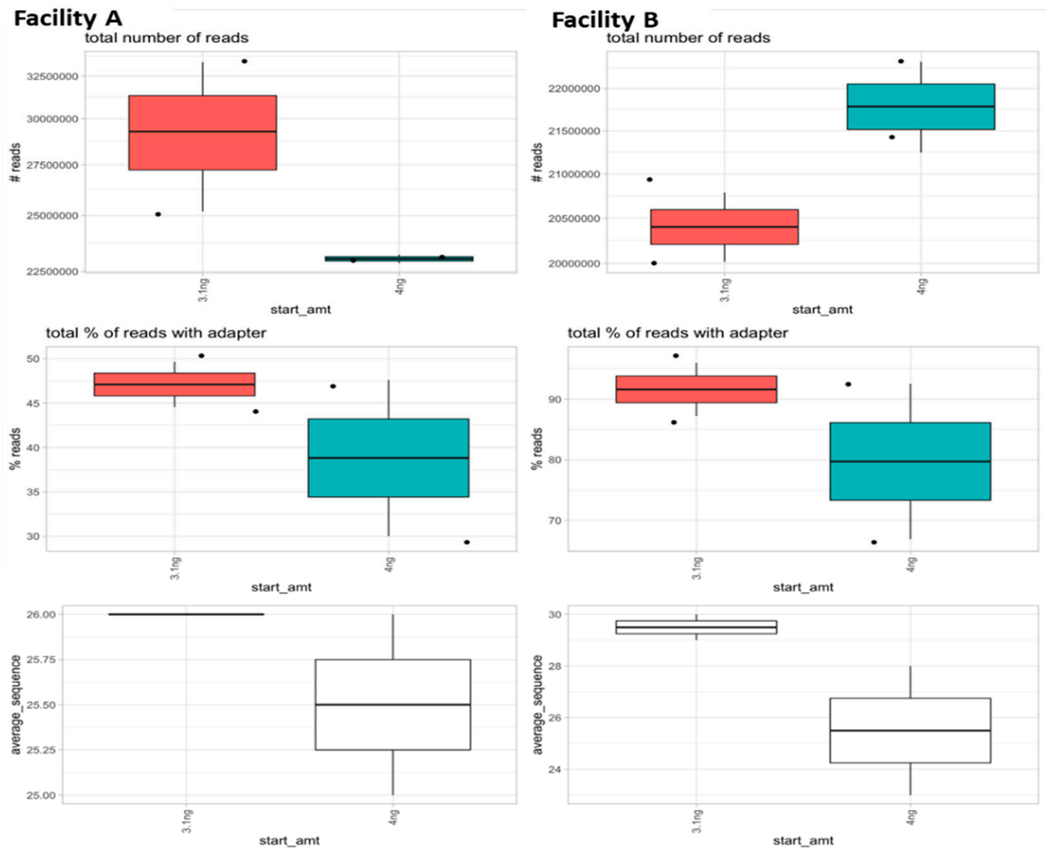

Supplemental Figure S5. QC analysis of sequencing data basic metrics from facility A and facility B. Comparison of the basic metrics between the two facilities: total number of reads (top), total % of reads with adapter (middle; *high number was expected here since the input fragments are short*), and average sequence length (bottom).

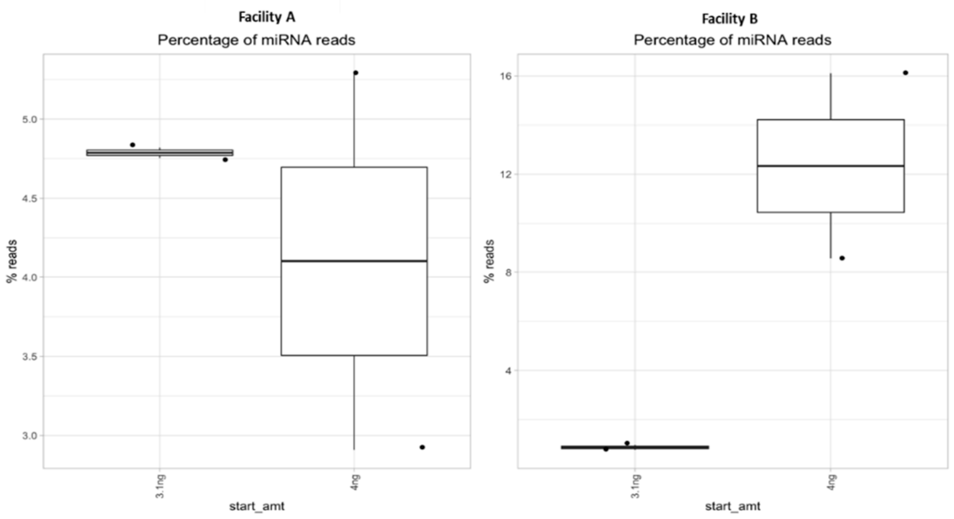

Supplemental Figure S6. Comparison of miRNA read percentages. *Note: Y-axes are scaled differently.*

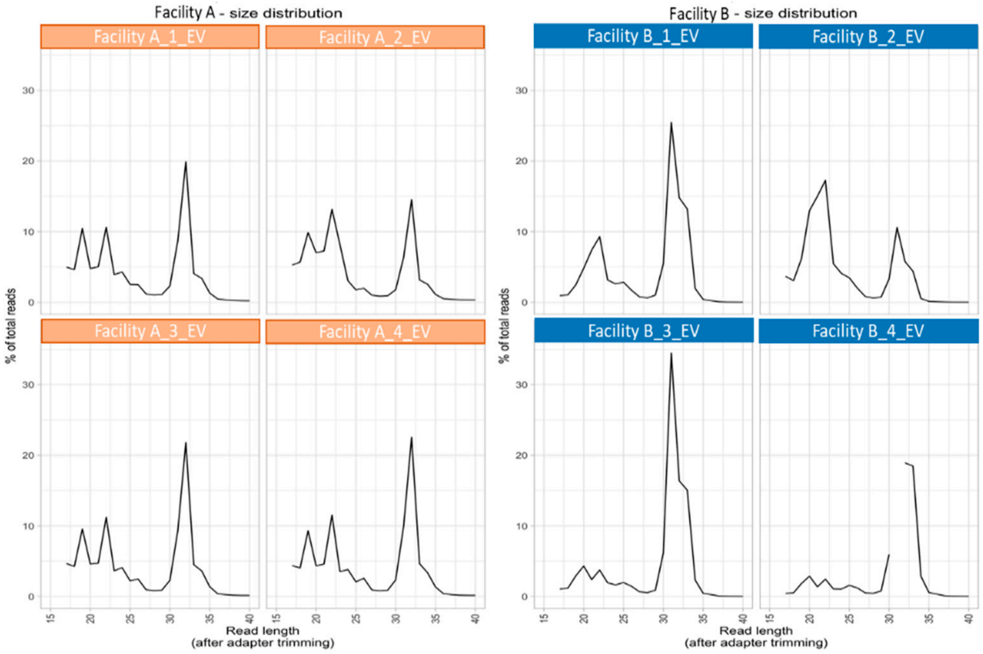

Supplemental Figure S7. Analysis of read size distribution of small RNA seq data from facility A and facility B. Size distribution comparison after adapter removal. The x-axes show the read length (nts), and the y-axes show the number of reads.

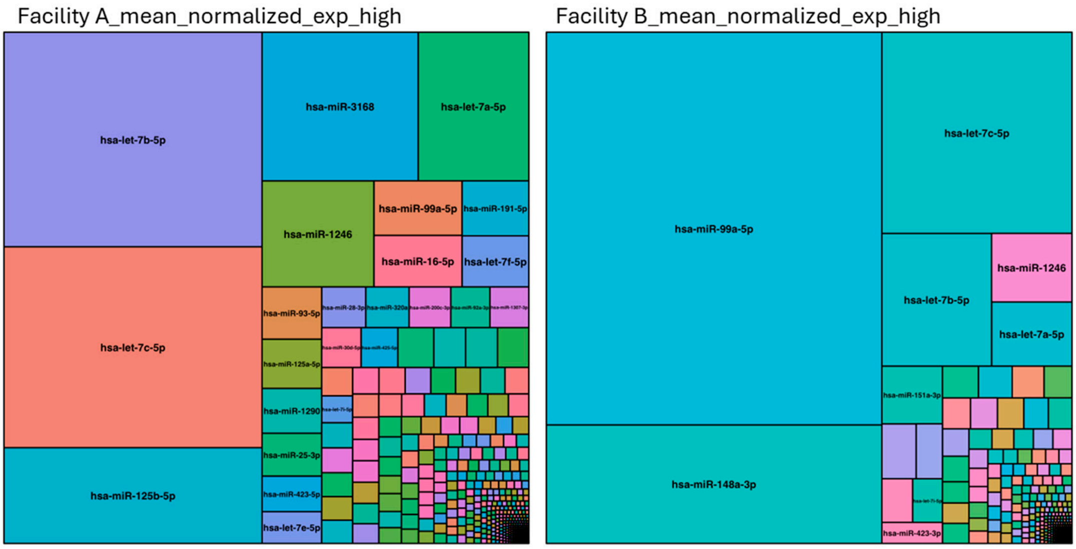

Supplemental Figure S8. Differential expression of miRNA identified from the two facilities in the analysis presented as treemaps for high content (4 ng) concentration. *Note: A complete list of each miRNA with differential expression profiles in a tabular format can also be found in the Supplemental Report (https://rpubs.com/snp34/1293230, accessed on 9 December 2025).*

Supplemental Code:

Although binning the continuous measurements into ordinal bins for the multinomial model removes some resolution, it does eliminate the need to assume a specific distribution when fitting to a continuous model. The multinomial model enables direct, standardized visual and statistical comparisons across methods and replicates.

```
#Example code script using R for data analysis

#lnc.pta.d1, lnc.pta.d2, and lnc.pta.d3
#are vectors of the diameter sizes from replicates 1, 2, and 3  of the LNCaP cell line using the PTA instrument that have been loaded from data spreadsheets

n.lnc.pta1=length(  lnc.pta.d1  )

n.lnc.pta2=length(lnc.pta.d2)

n.lnc.pta3=length(lnc.pta.d3)


lnc.pta.tops=65+(0:42)*5

####Binning function
Bins=function (x, boxtops, incr) {
 n= length(boxtops)
 bin.num=rep( NA,n)

for (i in 1:n){
 yes.inbox=  (x >= ( boxtops[i] -incr) ) & (  x <  boxtops[i]  )
```

```
 bin.num[i]=sum(yes.inbox)
 }
 return(list( boxtops=boxtops, bin.num=bin.num))}
#######

lnc.pta1.res= Bins( lnc.pta.d1,  lnc.pta.tops, 5)

lnc.pta2.res= Bins( lnc.pta.d2, lnc.pta.tops, 5)

lnc.pta3.res= Bins( lnc.pta.d3 , lnc.pta.tops, 5)


 empir.mean.lnc.pta1=    lnc.pta1.res$bin.num/n.lnc.pta1

 empir.mean.lnc.pta2=    lnc.pta2.res$bin.num/n.lnc.pta2

 empir.mean.lnc.pta3=    lnc.pta3.res$bin.num/n.lnc.pta3



 sigma.lnc.pta1=    sqrt(    (empir.mean.lnc.pta1)*(1-
empir.mean.lnc.pta1)/n.lnc.pta1 )

 sigma.lnc.pta2=    sqrt(    (empir.mean.lnc.pta2)*(1-
empir.mean.lnc.pta2)/n.lnc.pta2 )

 sigma.lnc.pta3=    sqrt(    (empir.mean.lnc.pta3)*(1-
empir.mean.lnc.pta3)/n.lnc.pta3 )

#####


empir.mean.lnc.pta.all=    (empir.mean.lnc.pta1    +
empir.mean.lnc.pta2 +empir.mean.lnc.pta3)/3


set.seed(1)
lnc.pta1.r    =rmultinom(n=1e5,size=n.lnc.pta1,    prob=
lnc.pta1.res$bin.num  )

lnc.pta1.rp= lnc.pta1.r / n.lnc.pta1


lnc.pta2.r    =rmultinom(n=1e5,size=n.lnc.pta2,    prob=
lnc.pta2.res$bin.num  )

lnc.pta2.rp= lnc.pta2.r / n.lnc.pta2

lnc.pta3.r    =rmultinom(n=1e5,size=n.lnc.pta3,    prob=
lnc.pta3.res$bin.num  )
```

```
lnc.pta3.rp= lnc.pta3.r / n.lnc.pta3

 lnc.pta.all.rp = cbind(   lnc.pta1.rp, lnc.pta2.rp,lnc.pta3.rp)

#uncert. of mean bin probs:

sd.lnc.pta.all.rp  = apply(lnc.pta.all.rp,1,sd)

##############

#######################################
```
